# In Vitro Generation of Novel Functionalized Biomaterials for Use in Oral and Dental Regenerative Medicine Applications. Running Title: Fibrin–Agarose Functionalized Scaffolds

**DOI:** 10.3390/ma13071692

**Published:** 2020-04-04

**Authors:** Cristina Blanco-Elices, Enrique España-Guerrero, Miguel Mateu-Sanz, David Sánchez-Porras, Óscar Darío García-García, María del Carmen Sánchez-Quevedo, Ricardo Fernández-Valadés, Miguel Alaminos, Miguel Ángel Martín-Piedra, Ingrid Garzón

**Affiliations:** 1 Department of Histology (Tissue Engineering Group), University of Granada, 18071 Granada, Spain; acblanco96@gmail.com (C.B.-E.); miguel.mateu@upc.edu(M.M.-S.); davidsp94@correo.ugr.es (D.S.-P.); oscargg@correo.ugr.es (Ó.D.G.-G.); mcsanchez@ugr.es (M.d.C.S.-Q.); rfdezvalades@me.com (R.F.-V.); malaminos@ugr.es (M.A.); 2 Programa de doctorado Medicina Clínica y Salud Pública, University of Granada, 18071 Granada, Spain; enrique@dentalos.es; 3 Department Materials Science and Metallurgy (Biomaterials, Biomechanics and Tissue Engineering Group), Technical University of Catalonia, 08019 Barcelona, Spain; 4 Department of Histology, Faculty of Medicine, University of Granada, Avenida de la Investigación 11, E18016, 18071 Granada, Granada, Spain; igarzon@ugr.es

**Keywords:** functionalization, oral and dental tissues, biomaterials, extracellular matrix, tissue engineering

## Abstract

Recent advances in tissue engineering offer innovative clinical alternatives in dentistry and regenerative medicine. Tissue engineering combines human cells with compatible biomaterials to induce tissue regeneration. Shortening the fabrication time of biomaterials used in tissue engineering will contribute to treatment improvement, and biomaterial functionalization can be exploited to enhance scaffold properties. In this work, we have tested an alternative biofabrication method by directly including human oral mucosa tissue explants within the biomaterial for the generation of human bioengineered mouth and dental tissues for use in tissue engineering. To achieve this, acellular fibrin–agarose scaffolds (AFAS), non-functionalized fibrin-agarose oral mucosa stroma substitutes (n-FAOM), and novel functionalized fibrin-agarose oral mucosa stroma substitutes (F-FAOM) were developed and analyzed after 1, 2, and 3 weeks of in vitro development to determine extracellular matrix components as compared to native oral mucosa controls by using histochemistry and immunohistochemistry. Results demonstrate that functionalization speeds up the biofabrication method and contributes to improve the biomimetic characteristics of the scaffold in terms of extracellular matrix components and reduce the time required for in vitro tissue development.

## 1. Introduction

Regenerative medicine applies multidisciplinary biology and engineering science to therapeutic approaches to regenerate, replace, or repair tissues and organs [1]. In the field of dentistry, regenerative medicine has been used for the treatment of complex diseases in which oral cavity and dental tissues are damaged or lost, including endodontic treatments [2], periodontics [3], and other disciplines. One of the most promising areas of regenerative medicine is tissue engineering, which combines human cells with biocompatible biomaterials or scaffolds in order to generate tissue substitutes able to promote tissue regeneration [4]. Although we have previously developed some promising models of human periodontal tissues by tissue engineering [5], most biomaterials are not able to provide adequate interaction with human cells due to their poor mechanical, chemical, and biological properties, and biofabrication of these bioartificial tissues may take long periods of time. Therefore, novel methods able to improve scaffolds for use in dental tissue engineering are in need.

In this regard, scaffold functionalization can be exploited as a novel method to improve the physiological characteristics of biomaterials by tailoring its surface in accordance to the physiological surrounding of the living cells [6]. Functionalization is based on the introduction of functional components inside or on the surface of the scaffold in order to enhance cell adhesion, proliferation and differentiation. The functionalization process can be performed by using chemical compounds able to enhance hydrophilicity of different biomaterials or other types of bioactive molecules providing relevant functions including cell recognition, proliferation and differentiation [7]. Scaffolds can be incorporated with bioactive molecules, such as extracellular matrix (ECM) proteins, drugs, peptide sequences, and growth factors that are capable of interact with cells when they are released. Some of the ECM proteins employed in functionalization process are collagen and glycosaminoglycans [8], fibronectin [9], laminin [10], or short peptide sequences derived from them that have major stability and interplay with cells [11]. These molecules can be added to scaffolds by different techniques, that includes physical absorption of a solution and covalent binding [12]. All these reported techniques are intended to separately introduce functional molecules in the biomaterial, so that these molecules can later interact with cells.

In this milieu, one of the main limitations of tissue engineering is the need of obtaining adequate primary cell cultures generated from small tissue biopsies, which are later combined with biomaterials to generate a tissue substitute. In oral cavity and dental tissue engineering, biopsies are typically obtained from patient’s oral mucosa, palate, periodontal tissue, or tooth, which are enzymatically digested to isolate an initial cell population that is subsequently expanded for further use in tissue engineering [13]. The fact that primary cell cultures show very low expansion and proliferation rate is one of the major factors associated to the long time required for the efficient generation of a human tissue substitute by tissue engineering [14] and supports the search for alternative strategies.

In the present work, we have tested an alternative biofabrication method for the generation of human bioengineered mouth and dental tissues for use in tissue engineering. This method allows scaffold functionalization and speeds up the biofabrication method by directly including human oral mucosa tissue explants within the biomaterial in order to enhance the physiological and biochemical properties of the scaffold. This technique could improve the biomimetic characteristics of the scaffold in terms of ECM components and reduce the time required for in vitro tissue development.

## 2. Materials and Methods

### 2.1. Generation of Acellular Fibrin–Agarose Scaffolds (AFAS)

Acellular fibrin–agarose biomaterials were generated as reported elsewhere [15,16]. First, normal human plasma obtained from healthy donors. Then, 15.2 mL of plasma were mixed with 1.5 mL of Dulbecco’s Modified Eagle’s Medium (DMEM), 2 mL of 1% CaCl_2_ and 1 mL of melted concentrated 2% type VII agarose (all of them from Sigma-Aldrich/Merck, St. Louis, MO, USA) and 300 µL of tranexamic acid (Amchafibrin, Fides Ecopharma, Valencia, Spain). This mixture was aliquoted in 128 × 86 mm culture dishes and kept at 37 °C in a cell incubator to promote jellification and DMEM culture medium containing 10% fetal bovine serum and antibiotics was added 24 h later and renewed every 3 days.

### 2.2. Generation of Non-Functionalized Fibrin–Agarose Oral Mucosa Stroma Substitutes (n-FAOM)

Previously reported human oral mucosa stroma substitutes based on fibrin–agarose biomaterials (n-FAOM) were generated following previously published methods [13,15]. Briefly, human oral mucosa biopsies were obtained from healthy donors subjected to minor oral surgery with local anesthesia at the School of Dental Sciences of the University of Granada, Spain. Immediately after extraction, biopsies were washed in phosphate-buffered saline (PBS) and sectioned in small explants with an average size of 0.5 × 0.5 × 0.5 mm, which were digested for 6–8 h in a 2 mg/mL solution of *Clostridium histolyticum* type-I collagenase (Gibco BRL, Waltham, MA, USA) at 37 °C. Isolated fibroblasts were recovered by centrifugation and cultured in Dulbecco’s modified Eagle’s minimal essential medium with 1% antibiotics and 10% fetal bovine serum (Sigma-Aldrich/Merck, St. Louis, MO, USA). Once cells were expanded, a fibrin–agarose biomaterial mixture was generated as reported for the AFAS, and 10,000 human oral fibroblasts per ml of mixture were trypsinized and added to the mixture just before inducing jellification. This mixture was aliquoted in 128 × 86 mm culture dishes and kept at 37 °C in a cell incubator to promote jellification and DMEM culture medium containing 10% fetal bovine serum and antibiotics was added 24 h later and renewed every 3 days.

The work was approved by the local research committee (Comité Coordinador de Ética de la Investigación Biomédica de Andalucía, ref. 0116-N-19), and all patients provided written consent to participate in the study.

### 2.3. Generation of Functionalized Fibrin–Agarose Oral Mucosa Stroma Substitutes (F-FAOM)

To generate functionalized substitutes, 3 × 3 × 3 mm human oral mucosa biopsies were obtained from healthy donors undergoing minor oral surgery. Immediately after extraction, all tissues were kept at 4 °C in a transport medium (Dulbecco’s modified Eagle’s medium DMEM; 100 U/mL of penicillin G (Sigma-Aldrich, Burlingame, CA, USA), 100 μg/mL of streptomycin (Sigma-Aldrich, Burlingame, CA, USA), and 0.25 μg/mL of amphotericin B (Sigma-Aldrich, Burlingame, CA, USA) and processed in the following 24 h. Then, tissue specimens were washed in phosphate-buffered saline (PBS) and culture medium. The tissue specimens were then cut into small pieces (around 0.5 × 0.5 × 0.5 mm in size), which were resuspended as tissue explants in 500 μL of DMEM. Then, this suspension was combined with the fibrin-agarose mixture generated as explained above, before inducing jellification of the biomaterial. Briefly, 15.2 mL of human plasma were mixed with 1 mL of DMEM, and the 500 μL suspension of the explants in DMEM. Then, 300 µL of tranexamic acid, 2 mL of 1% CaCl_2_ and 1 mL of 2% type VII agarose were added, the mixture was carefully mixed and aliquoted in 128 × 86 mm culture dishes and kept at 37 °C in a cell incubator to promote jellification. DMEM culture medium containing 10% fetal bovine serum and antibiotics was added 24 h later and renewed every 3 days.

### 2.4. Histological Analyses

AFAS, n-FAOM, and F-FAOM samples were fixed in 3.7–4.0% w/v formaldehyde buffered to pH 7 and stabilized with methanol for 24 h at 4 °C, dehydrated, and embedded in paraffin. Tissue sections of 5 µm of thickness were obtained, placed on glass slides, and dried at room temperature. Samples were then dewaxed in xylene, cleared, and rehydrated, and stained with hematoxylin and eosin (H&E) (PanReac Química S.L.U., Barcelona, Spain) and histologically analyzed using a Nikon Eclipse 90i light microscope. Images were captured using a Nikon RS-Di2 camera with NIS-Elements BR 4.50.00 64-bit software (all from Nikon Corp., Tokyo, Japan). All tissues (AFAS, n-FAOM and F-FAOM) were kept in culture for up to 3 weeks, and samples were taken for analysis after 1, 2 and 3 weeks of in vitro development. This time period was selected since this was the moment in which fibrin-agarose bioengineered human oral mucosa demonstrated the highest levels of in vitro maturation and could be grafted in vivo in previous works [5,17,18]. Normal human oral mucosa was used as control (CTR samples). 

### 2.5. Analysis of ECM Components by Histochemistry

The main components of the human mouth and dental tissues ECM were detected by histochemistry in AFAS, n-FAOM, and F-FAOM and normal oral mucosa controls. First, paraffin-embedded tissue sections were obtained, dewaxed, and rehydrated with distilled water. Then, Picrosirius red and Gomori histochemical methods were performed for detection of mature collagen and reticular fibers, respectively, whereas the histochemical methods of alcian blue and periodic acid-Schiff (PAS) were applied to identify proteoglycans and glycoproteins, respectively. For Picrosirius red, samples were stained with a Sirius red F3B working solution for 30 min and counterstained with Harris Hematoxylin for 5 min. To detect reticular fibers, Gomori staining was performed using 1% potassium permanganate, 2% sodium metabisulphite, and 2% iron solution. Subsequently, ammoniacal silver and 20% formaldehyde was used prior to differentiation with gold chloride and thiosulphate. For Alcian Blue, samples were stained in Alcian Blue reagent solution for 30 min to stain tissue proteoglycans. For the PAS method, tissue sections were incubated in a 0.5% periodic acid solution for 5 min as oxidant, followed by incubation in Schiff reagent for 15 min and counterstaining with Harris hematoxylin for 1 min (all reagents were from PanReac Química S.L.U., Barcelona, Spain). Normal human tissues were used as technical controls for each method.

Quantitative analysis of ECM components in each tissue type was performed by determining the signal intensity found for each histochemical method in each sample (AFAS, n-FAOM, and F-FAOM after 1, 2, and 3 weeks of culture and oral mucosa controls). For this, images were analyzed using the ImageJ 1.51n software as previously reported [19], and average signal intensity was calculated in each case. In short, 10 points were randomly selected in each image and the staining intensity was automatically assessed by the software, and subtracted to the background blank signal. Finally, the signal was referred to the control native oral mucosa considered as 100% signal. All analyses were carried out using 10 independent samples (n = 10).

### 2.6. Immunohistochemical Analysis

To identify the presence of mature and immature type-I collagen fibers in the different tissue types, we used immunohistochemistry for type-I collagen epitopes. In addition, stromal fibroblast cells were detected in the stroma of each tissue by immunohistochemical labeling of vimentin. Briefly, tissue sections were dewaxed and rehydrated and citrate buffer was used at 98 °C for 25 min for antigen retrieval. Samples were incubated in 3% H_2_O_2_ for 10 min to quench endogenous peroxidase activity. Each of the succeeding steps was followed by a thorough rinse in PBS and were performed in a humid chamber to prevent tissue desiccation. Non-specific staining was blocked with a normal horse serum solution (NHS) (Vector Laboratories, Burlingame, CA) for 15 min. Sections were incubated with the primary antibodies diluted in NHS using a 1:200 dilution for anti-type-I collagen (Acris/OriGene Technologies GmbH, Herford, Germany) and anti-vimentin antibodies (Sigma-Aldrich). As a secondary antibody, samples were incubated in ImmPRESS^TM^ HRP Reagent Kit Universal anti-mouse/rabbit IgG solution (Vector Laboratories). Subsequently, peroxidase reaction was visualized using a peroxidase substrate kit DAB SK-4011 (Vector Laboratories). The reaction was immediately interrupted using tap water after the reaction occurs. Then, sections were counterstained with Harris hematoxylin (PanReac Química S.L.U.) for 15s. Native human control tissues were used as technical controls.

In the case of type-I collagen, images were taken from each tissue and the signal intensity was quantitatively determined as describe for the histochemical methods. For vimentin, the number of positive cells per unit of area was counted in each tissue type (AFAS, n-FAOM and F-FAOM after 1, 2 and 3 weeks of culture and oral mucosa controls). All analyses were carried out using 10 independent samples (n = 10).

### 2.7. Statistical Analyses

In the first place, we determined the average value and standard deviation of quantitative values obtained for each histochemical and immunohistochemical analysis for each of the following study groups:

(1) Global groups of samples. Global groups corresponded to each type of tissue, regardless the development time: oral mucosa controls (CTR), fibrin-agarose scaffolds (AFAS), non-functionalized fibrin-agarose oral mucosa stroma substitutes (n-FAOM), and fibrin-agarose oral mucosa stroma substitutes (F-FAOM).

(2) Specific samples. These groups corresponded to each type of tissue at each specific in vitro development time: CTR, AFAS corresponding to 1, 2, and 3 weeks of development (AFAS-1W, AFAS-2W, and AFAS-3W, respectively); n-FAOM corresponding to 1, 2, and 3 weeks of development (n-FAOM-1W, n-FAOM-2W, and n-FAOM-3W, respectively); and F-FAOM corresponding to 1, 2, and 3 weeks of development (F-FAOM-1W, F-FAOM-2W, and F-FAOM-3W, respectively).

All these values were normalized to the values found in CTR samples, which were considered as 100% intensity for each analysis method.

In the second place, we evaluated the statistical significance of differences among groups of samples using the ANOVA test with Tukey post hoc analysis for pairwise comparisons between two study groups. In all cases, we compared the global groups of samples to identify global differences among these groups, and the specific samples to identify differences among specific types of samples. Analysis of correlation between different parameters (histochemical and immunohistochemical methods) was performed with Kendall–Tau statistical test. A Bonferroni-adjusted significance level of 0.0001 was considered for statistically significant values, since up to 435 statistical tests were performed in this work. In turn, *p*-values between 0.05 and 0.0001 were considered as marginally significant. To carry out this statistical analysis, we used SPSS 15.0 software.

## 3. Results

### 3.1. Histological Analysis of Functionalized and Non-Functionalized Fibrin–Agarose Oral Mucosa Stroma Substitutes

**Figure 1:**
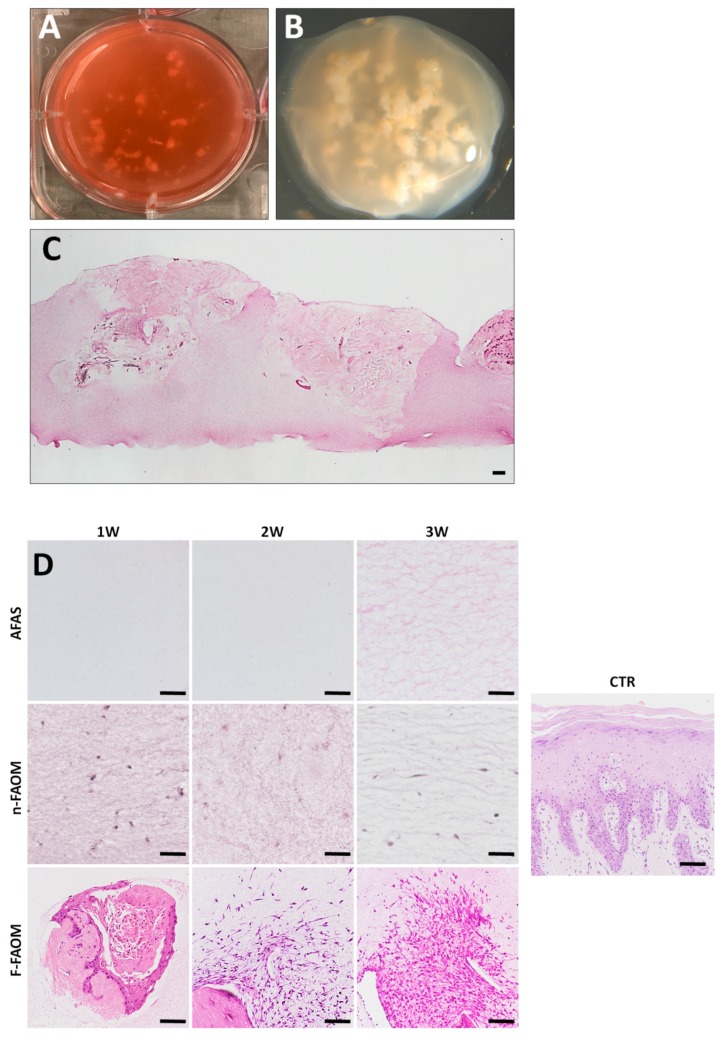
In vitro development of functionalized fibrin–agarose oral mucosa substitutes (F-FAOM). (**A**,**B**) Macroscopic images of functionalized F-FAOM. (**C**) Histological evaluation of F-FAOM stained with hematoxylin eosin. Scale bar: 100 µm. Analysis of controls and biofabricated oral tissues using hematoxylin and eosin staining (**D**). CTR: control native oral mucosa; AFAS: acellular fibrin–agarose scaffolds; n-FAOM: non-functionalized fibrin-agarose oral mucosa stroma substitutes using isolated primary cell cultures of human oral mucosa fibroblasts immersed in fibrin–agarose scaffolds; F-FAOM: functionalized fibrin–agarose oral mucosa stroma substitutes using oral mucosa tissue explants directly immersed in fibrin–agarose scaffolds. 1W: samples kept in vitro 1 week; 2W: samples keep in vitro 2 weeks; 3W: samples keep in vitro 3 weeks. Scale bars: 100 μm.

First, gross inspection of the bioartificial tissues generated by using the functionalization method described in the present work showed a compact biomaterial in which the tissue explants were immersed (Figure 1A,B), whereas AFAS and n-FAOM samples were apparently more regular and homogeneous due to the lack of explants immersed within. No gross macroscopic differences were observed with development time (1 to 3 weeks) for any of the three sample types.

Then, the histological analysis using H&E revealed that the oral mucosa explants were completely surrounded by the fibrin–agarose scaffold and properly integrated in the biomaterial (Figure 1C), whereas the other tissue types (AFAS and n-FAOM) consisted of a fibrillar biomaterial with a regular structure that only contained cells in the case of n-FAOM tissues. As shown in Figure 1D, we found important differences among sample types and among development times. Analysis of samples corresponding to 1 week of in vitro development showed that AFAS were characterized by a homogenous fibrillary network devoid of cells, whereas n-FAOM consisted on a fibrillary network containing a fibroblast cell population with a spindle-shape, elongated morphology suggesting that cells are properly integrated in the mesh. In contrast, analysis of F-FAOM after 1 week of development revealed a heterogeneous mixture of the native oral mucosa explants immersed within the biomaterial, which was regular and did not have isolated cells growing on it. When samples corresponding to 2 and 3 weeks of development were analyzed, we found significant differences for F-FAOM, but not for AFAS or n-FAOM (Figure 1D). In the case of the AFAS and n-FAOM samples, the histological structure of the fibrillar mesh and the cell population (for n-FAOM) showed very few changes with time, suggesting that these tissues tended to remain stable with the development time. However, samples corresponding to 2 weeks of development of F-FAOM tissues showed that the cells tended to migrate grow and proliferate from the explants towards the biomaterial fibrillar mesh. This trend continued over time and 3 weeks F-FAOM samples had a more abundant cell population hosted by the fibrin–agarose biomaterial surrounding the explants. No signs of cell death, necrosis or any other alterations were morphologically detected in any of the samples. In turn, control native oral mucosa stroma showed abundant stromal cells immersed in the stroma ECM and showed the typical papillae interdigitating with the epithelial layer (Figure 1D).

### 3.2. Cell Proliferation in the Fibrin–Agarose Biomaterial

**Figure 2:**
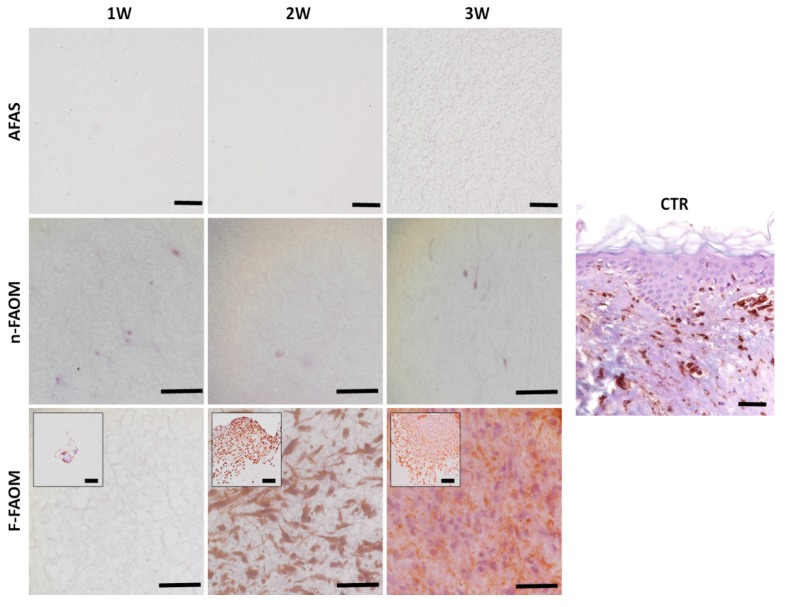
Immunohistochemical analysis of controls and biofabricated oral tissues using vimentin primary antibodies. CTR: control native oral mucosa; AFAS: acellular fibrin–agarose scaffolds; n-FAOM: non-functionalized fibrin–agarose oral mucosa stroma substitutes using isolated primary cell cultures of human oral mucosa fibroblasts immersed in fibrin-agarose scaffolds; F-FAOM: functionalized fibrin–agarose oral mucosa stroma substitutes using oral mucosa tissue explants directly immersed in fibrin–agarose scaffolds. 1W: samples kept in vitro 1 week; 2W: samples keep in vitro 2 weeks; 3W: samples keep in vitro 3 weeks. Scale bars: 100 μm.

As expected, analysis of the vimentin-positive stromal cells within each type of fibrin–agarose biomaterial first showed no cells in AFAS. Then, we found that n-FAOM global group contained an average of 292 ± 38 cells per mm^2^ of bioartificial tissue, and that this cell population tended to remain stable with time (325 ± 125, 250 ± 173, and 300 ± 81 cells per mm^2^ of bioartificial tissue in n-FAOM-1W, n-FAOM-2W, and n-FAOM-3W, respectively). Finally, we found that F-FAOM global samples showed an average number of 3167 ± 2813 cells, which was significantly higher than n-FAOM global group (*p* < 0.0001), but similar to CTR (*p* > 0.005). For the F-FAOM specific samples, we found 0 ± 0, 4125 ± 457, and 5375 ± 419 cells per mm^2^ of bioartificial tissue in F-FAOM-1W, F-FAOM-2W, and F-FAOM-3W, respectively, with F-FAOM-3W being statistically higher than the rest of samples (*p* < 0.0001). As shown in Figure 1D and Figure 2, we did not find any cells outside the explants and within the biomaterial in the F-FAOM-1W group, but the number of cells found in the biomaterial tended to increase with time from the second week of development, with a positive significant correlation between the cell number and the culture time (r = 0.4961, *p* < 0.0001). Although cells did not have time to migrate from the explants at week 1, cells migrated and proliferated into the scaffold in F-FAOM samples from the second week onwards. In addition, the cell number correlated in a positive and significant way with the amount of proteoglycans (r = 0.6027; *p* < 0.0001), glycoproteins (r = 0.5227; *p* < 0.0001), and collagen as determined by Picrosirius red histochemistry (r = 0.4134; *p* < 0.0001) and immunohistochemistry (r = 0.4439; *p* < 0.0001).

### 3.3. Development of Non-Fibrillar ECM Components in the Fibrin–Agarose Biomaterial

**Figure 3:**
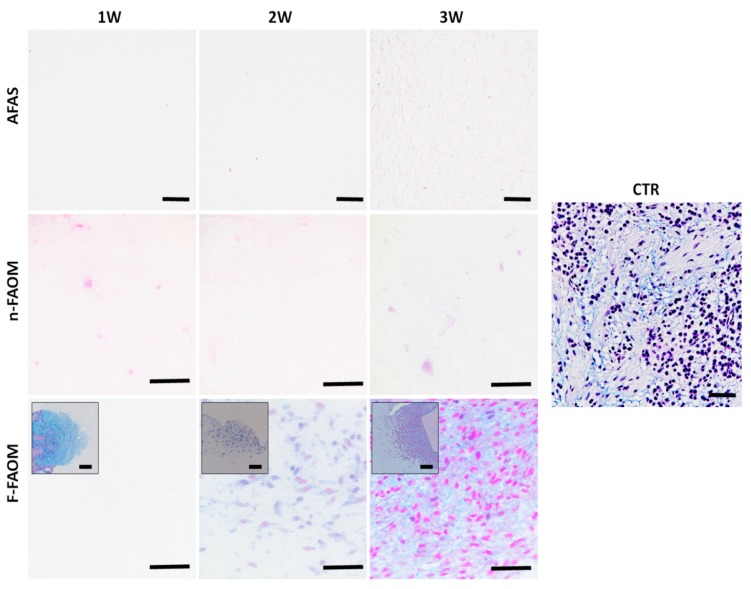
Histochemical analysis of controls and biofabricated oral tissues using Alcian Blue staining. CTR: control native oral mucosa; AFAS: acellular fibrin–agarose scaffolds; n-FAOM: non-functionalized fibrin–agarose oral mucosa stroma substitutes using isolated primary cell cultures of human oral mucosa fibroblasts immersed in fibrin–agarose scaffolds; F-FAOM: functionalized fibrin–agarose oral mucosa stroma substitutes using oral mucosa tissue explants directly immersed in fibrin–agarose scaffolds. 1W: samples kept in vitro 1 week; 2W: samples keep in vitro 2 weeks; 3W: samples keep in vitro 3 weeks. Scale bars: 100 μm.

To determine the suitability of each type of bioartificial tissue to properly generate the main components of the human oral mucosa ECM, we first analyzed the presence of proteoglycans using alcian blue histochemical staining (Figure 3). Results (Table 1) showed that both the acellular AFAS and n-FAOM global groups were mostly devoid of these ECM components, with differences among both being statistically non-significant (*p* > 0.05). In contrast, samples corresponding to the F-FAOM global group showed almost twice the proteoglycans expression found in AFAS and n-FAOM, although differences with these two global groups were only marginally significant (*p* = 0.0020 vs. AFAS and *p* = 0.0010 vs. n-FAOM). However, the level of proteoglycans expression in F-FAOM did not reach the high levels found for the control native oral mucosa samples (differences were statistically significant, *p* < 0.0001). When specific groups of samples were analyzed, we found that F-FAOM-3W had significantly higher proteoglycans content than acellular and non-functionalized samples (AFAS-1W, AFAS-2W, AFAS-3W, n-FAOM-1W, n-FAOM-2W, and n-FAOM-3W), and F-FAOM-1W, being marginally higher than F-FAOM-2W (*p* = 0.0060), and marginally lower than CTR (*p* = 0.0010). Correlation with culture time was positive and statistically significant (r = 0.5020; *p* < 0.0001) (Supplementary Table S1).

**Figure 4:**
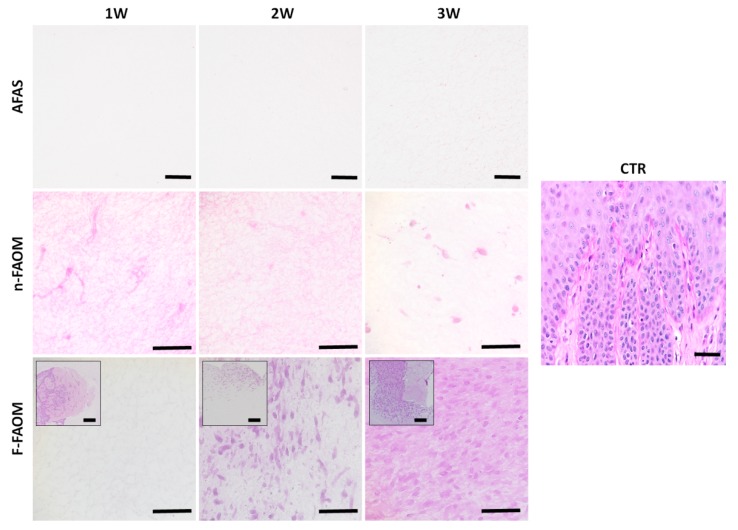
Histochemical analysis of controls and biofabricated oral tissues using PAS staining. CTR: control native oral mucosa; AFAS: acellular fibrin–agarose scaffolds; n-FAOM: non-functionalized fibrin–agarose oral mucosa stroma substitutes using isolated primary cell cultures of human oral mucosa fibroblasts immersed in fibrin-agarose scaffolds; F-FAOM: functionalized fibrin–agarose oral mucosa stroma substitutes using oral mucosa tissue explants directly immersed in fibrin–agarose scaffolds. 1W: samples kept in vitro 1 week; 2W: samples keep in vitro 2 weeks; 3W: samples keep in vitro 3 weeks. Scale bars: 100 μm.

In the second place, we analyzed the synthesis of ECM glycoproteins by using the PAS method (Figure 4). Results showed that the content of glycoproteins in AFAS and n-FAOM global groups was very low (significantly lower than CTR in both cases, *p* < 0.0001). However, F-FAOM and CTR showed significantly higher contents of these ECM components, although F-FAOM samples were significantly lower than CTR. For the specific groups of samples, we found very low PAS staining intensity in all acellular and non-functionalized samples, as well as in F-FAOM-1W. However, F-FAOM-2W and F-FAOM-3W samples showed significantly higher intensity than acellular and non-functionalized samples and F-FAOM-1W, with marginally significant differences between F-FAOM-2W and F-FAOM-3W (*p* = 0.0001). In addition, F-FAOM-3W samples were statistically comparable to CTR samples (*p* > 0.05). Correlation with the time in culture was statistically non-significant (*p* > 0.05), but results found for alcian blue significantly correlated in a positive way with PAS results (r = 0.3276; *p* < 0.0001) (Supplementary Table S1).

### 3.4. Development of Fibrillar ECM Components in the Fibrin–Agarose Biomaterial

Histochemical analysis of reticular fibers as determined by Gomori staining revealed that all samples (including CTR) were devoid of these fibers, and no differences were found among CTR, AFAS, n-FAOM, and F-FAOM samples (data not shown).

**Figure 5:**
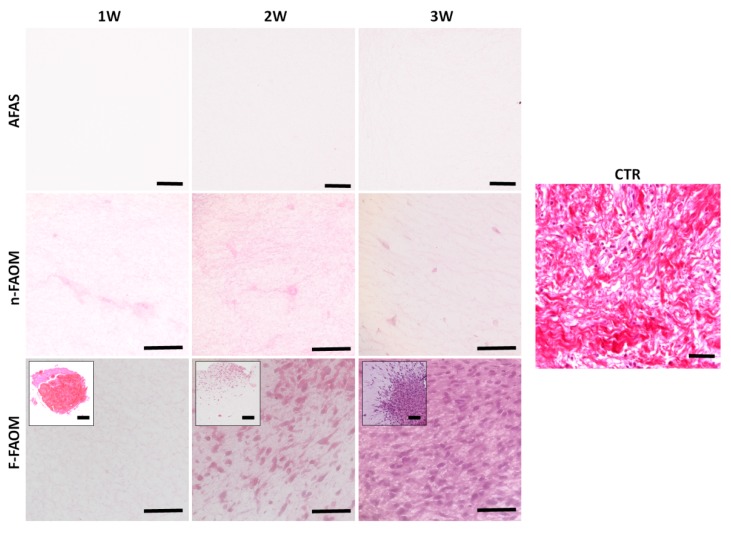
Histochemical analysis of controls and biofabricated oral tissues using Picrosirius red staining. CTR: control native oral mucosa; AFAS: acellular fibrin–agarose scaffolds; n-FAOM: non-functionalized fibrin–agarose oral mucosa stroma substitutes using isolated primary cell cultures of human oral mucosa fibroblasts immersed in fibrin-agarose scaffolds; F-FAOM: functionalized fibrin–agarose oral mucosa stroma substitutes using oral mucosa tissue explants directly immersed in fibrin–agarose scaffolds. 1W: samples kept in vitro 1 week; 2W: samples keep in vitro 2 weeks; 3W: samples keep in vitro 3 weeks. Scale bars: 100 μm.

However, our analysis using Picrosirius red histochemistry showed a high concentration of mature collagen fibers in CTR human oral mucosa and a variable amount of these fibers in the rest of samples (Figure 5). Specifically, AFAS and n-FAOM global groups showed very low Picrosirius red staining intensity, with non-significant differences between them (*p* > 0.05), whereas F-FAOM showed more than twice the intensity level found in AFAS and n-FAOM (*p* < 0.0001). However, F-FAOM Picrosirius red staining expression was approximately half of the signal found in CTR, with differences being statistically significant (*p* < 0.0001). For specific groups, all samples were statistically lower than CTR, except for F-FAOM-3W, which had more nearly 85% of the staining intensity found in CTR and was statistically similar to this group (*p* < 0.05). Correlation of Picrosirius red staining with time was positive and statistically significant (r = 0.3503; *p* < 0.0001) (Supplementary Table S1).

**Figure 6:**
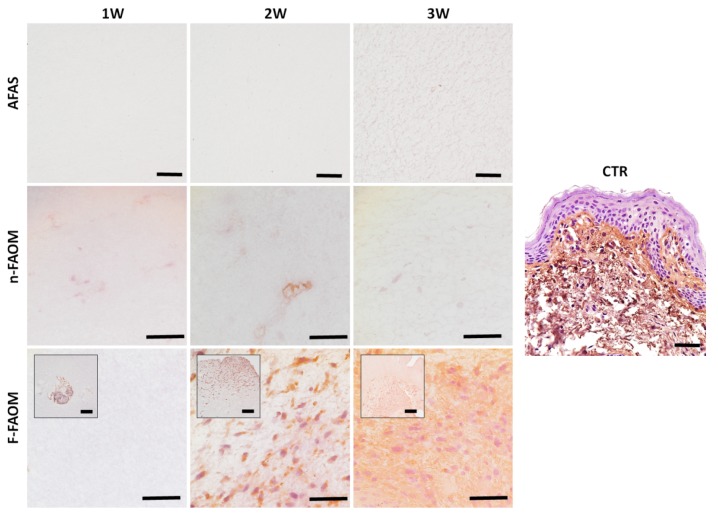
Immunohistochemical analysis of controls and biofabricated oral tissues using type-I collagen primary antibodies. CTR: control native oral mucosa; AFAS: acellular fibrin–agarose scaffolds; n-FAOM: non-functionalized fibrin–agarose oral mucosa stroma substitutes using isolated primary cell cultures of human oral mucosa fibroblasts immersed in fibrin–agarose scaffolds; F-FAOM: functionalized fibrin–agarose oral mucosa stroma substitutes using oral mucosa tissue explants directly immersed in fibrin-agarose scaffolds. 1W: samples kept in vitro 1 week; 2W: samples keep in vitro 2 weeks; 3W: samples keep in vitro 3 weeks. Scale bars: 100 μm.

Finally, the immunohistochemical analysis of mature and immature collagen (Figure 6) confirmed that the highest concentration of type-I collagen corresponded to CTR samples, with statistical differences with AFAS, n-FAOM and F-FAOM. As found for Picrosirius red, AFAS and n-FAOM global groups had very low amount of collagen, with non-significant differences between them (*p* > 0.05), whereas F-FAOM was marginally higher than AFAS (*p* = 0.0004) and similar to n-FAOM (*p* > 0.05). Interestingly, F-FAOM-3W samples showed significantly higher immunostaining intensity than acellular tissues and F-FAOM-1W (*p* < 0.0001) and marginally higher intensity than non-functionalized samples and F-FAOM-2W. Correlation with time was positive, but only marginally significant (r = 0.2913; *p* = 0.0005).

Results found for the immunohistochemical analysis of type-I collagen significantly correlated in a positive way with those obtained for the Picrosirius red histochemical analysis (r = 0.4919; *p* < 0.0001), and also with alcian blue (r = 0.4012 *p* < 0.0001) and PAS (r = 0.4279; *p* < 0.0001) (Supplementary Table S1).

## 4. Discussion

In the present work, we have developed novel bioengineered tissues for use in oral and dental tissue engineering using novel functionalization methods based on human tissue explants. From a technical standpoint, we found that novel F-FAOM could be easily generated in the laboratory by combining small oral mucosa biopsies with fibrin–agarose biomaterials previously used in tissue engineering [17,18,20], without the need of establishing primary cell cultures previously. Non-functionalized fibrin–agarose biomaterials have previously shown adequate biomechanical properties [16] and very good in vivo biocompatibility in animal models [13,21], which allowed us to use these biomaterials in patients [22,23] with promising preliminary results. However, it is well known that previous non-functionalized bioartificial periodontal tissue models typically require several weeks of culture to obtain adequate numbers of stromal cells, followed by a maturation time of the bioartificial tissue of ~3 weeks once the tissue construct is generated [5]. In contrast, the novel biofabrication method described here was able to generate a functionalized biomaterial in a total of 3 weeks since obtaining the biopsy, which could significantly accelerate the biofabrication process and contribute to clinical translation. 

Other biofabrication processes able to enhance hard and soft tissue regeneration have been described in literature, such as self-assembling peptides, synthetic polymers, ceramic scaffolds, and composites [24]. Our functionalized biomaterial, however, combines fibrin—a natural, highly biocompatible biomaterial—with agarose, a biomaterial with better biomechanical properties [16] and explant functionalization, in order to achieve a bioartificial tissue with enhanced biological properties in regenerative medicine. 

When the number of stromal cells was analyzed, we found that cells were capable of actively proliferate in the F-FAOM, and the number of cells found after 3 weeks of culture was similar to native oral mucosa, and very different to n-FAOM. The reason why cells tend to proliferate more actively in F-FAOM than in n-FAOM remains unclear. Moreover, it has been proved that trypsin-EDTA and other digestion enzymes may induce damage of human cell membranes [25]. Therefore, we may hypothesize that the need of using enzyme digestion-based protocols in n-FAOM could exert critical cell damage and stress during the first weeks of culture, which could in turn, reduce cell proliferation during the first weeks. The fact that the novel functionalization method described here does not require a previous enzymatic digestion step has another important advantage over other methods. In most cases, these enzymes have animal origin, meaning that their use is associated to important regulatory concerns from a translational standpoint [26]. In contrast, this is one of the few methods described in oral and dental tissue engineering that does not require a previous cell culture step using enzymes of animal origin.

On the other hand, we found that functionalization was able to generate novel bioengineered tissues containing significant amounts of key ECM components with an important biological role. The ECM plays very important roles in cell physiology, cell communication, division, and differentiation, allowing cell nutrition and oxygen interchange in artificial tissues [27]. Despite its importance, most bioartificial tissues generated in the laboratory have very limited amounts of most ECM components while kept in vitro [14,17], and our results with AFAS and n-FAOM are in agreement with this statement. In contrast, the use of functionalization methods succeeded in generating relevant ECM components in a better scale as compared to non-functionalized biomaterials.

One of the most important components of the ECM is the non-fibrillar components, which are essential for the maintenance of the 3D spatial structure and hydration level of tissues [28]. Proteoglycans appear to be essential for several tissue functions, including regulation of protease activity, cellular response to growth factors, cell–cell and cell–matrix interaction, and collagen fibrillogenesis [29]. The use of functionalization methods was significantly associated to an increase of proteoglycans, and F-FAOM cultured for 3 weeks was only marginally different to control human oral mucosa biopsies. In addition, ECM glycoproteins are essential components of functional tissues and perform critical architectural and structural roles in native tissues [30]. The fact that n-FAOM tissues were virtually devoid of these ECM components is in agreement with our previous findings, suggesting that bioartificial tissues kept in vitro are not able to synthetize large amounts of ECM components in this stage of development [20]. In contrast, F-FAOM did show higher expression of ECM glycoproteins identified by PAS staining, and the amount of glycoproteins in F-FAOM was significantly higher than non-functionalized tissues, especially for F-FAOM-3W. This increased synthesis of non-fibrillar components of the ECM in F-FAOM could enhance tissue functionality once grafted in vivo as compared to bioartificial tissues with lower levels of differentiation.

The fact that we found a significantly higher amount of non-fibrillar ECM components in F-FAOM as compared to n-FAOM could be explained by two reasons: First, the native oral mucosa explants incorporated in the biomaterial could be able to release some of its soluble ECM components, which could in turn diffuse into the biomaterial fibrillar structure. Second, the increasing number of proliferating cells inside the biomaterial could be responsible of synthetizing and excreting abroad the ECM components found in the functionalized scaffold, which is supported by the positive correlation found between cell number and ECM components, and also between time of development and ECM synthesis. Although a combined mechanism is likely, future works should determine the origin of these components. In addition, recent works reported the use of biomaterials enriched in human cell exosomes, and exosome-enriched natural and synthetic scaffolds demonstrated to provide internal and external modulation factors able to enhance regeneration of dental tissues [31]. These findings point out the possibility that exosomes released from the tissue explants could play a beneficial role on development, maturation, and differentiation of the fibrin-garose biomaterial used in our functionalized tissue model.

Regarding the ECM fibrillar components, development of a well-structured 3D fibers mesh is crucial for cell differentiation and migration within tissues [28], and it is the main aspect responsible for the biomechanical properties of human stromal tissues [32]. In the first place, we confirmed that native human oral mucosa is very rich in type-I collagen and mature collagen fibers, but does not have detectable levels of reticular fibers, as previously demonstrated [17]. Then, we found that bioartificial tissues also lack expression of reticular fibers, but showed some collagen expression. Specifically, non-functionalized tissues had very limited expression of mature collagen fibers identified by Picrosirius red, although some levels of type-I collagen were found in low amount. These results are in agreement with our previous findings showing that collagen formation is very low in bioartificial tissues kept in vitro, especially in the case of mature collagen, and in vivo grafting is necessary for a full differentiation and significant collagen synthesis [13,17]. However, functionalized tissues were able to synthetize higher amounts of mature collagen, and F-FAOM-3W reached the intensity found in CTR samples, although results obtained for the immunohistochemical analyses were lower than those obtained for Picrosirius red histochemistry. The fact that CTR samples have very large amounts of mature and immature collagen fibrils along with abundant mature fibers could partially explain this difference. 

Another important finding of the present study is the correlation of most parameters with development time. Specifically, we found that the levels of proteoglycans and collagen fibers significantly increased with time. These results support the idea that the increasing number of cells may be the responsible, at least in part, of the sequential synthesis of ECM components in the biomaterial, and suggest that culture of functionalized bioartificial tissues for longer periods of time could succeed in generating tissues with higher amounts of these components.

In summary, in the present work, we describe a novel straightforward functionalization method allowing the efficient generation of F-FAOM. Analysis of functionalized biomaterials revealed that these bioartificial tissues are more biomimetic than non-functionalized tissues in terms of cell number and synthesis of ECM components, which supports their clinical use in a more rapid period of time. One of the possible applications of the functionalized biomaterials generated in this work is the generation of biological membranes for periodontal regeneration. Other potential applications could be replacement of the diseased or damaged oral mucosa in oral and maxillofacial medicine, and treatment of patients with palatal defects such as cleft palate. Although we have previously developed and described several potential models of bioengineered periodontal tissue [33], oral mucosa [14], and palate mucosa [13] functionalization could provide increased functional properties and therefore, better clinical outcomes in the future. 

Strengths of the present work include the use of human bioengineered tissues generated by using state-of-the-art biofabrication methods, and the number of histological, histochemical and immunohistochemical analyses carried out in a single work. However, one of the limitations of the present study is the need of performing all quality control analyses required by national Medicines Agencies for clinical translation of advanced therapies medicinal products generated by tissue engineering, including biomechanical, biodegradation, porosity, vascularization, and biocompatibility characterization once grafted in vivo. The fact that the basic biomaterial used in the present work fulfilled all these requirements and is currently approved for clinical use [22,23] supports the future clinical usefulness of F-FAOM, but future in vivo analyses should determine the real potential of these novel functionalized tissues. Another limitation is the lack of long-term follow-up, as tissues were analyzed up to 3 weeks. Again, further research is necessary to fully characterize these tissues and establish their clinical potential.

## Figures and Tables

**Table 1:** Quantitative evaluation of proteoglycans, glycoproteins, mature collagen fibers and type-I collagen as determined by alcian blue, PAS, Picrosirius red Histochemistry, and type-I collagen immunohistochemistry, respectively. Values correspond to average ± standard deviation of the staining intensity found for each sample type normalized to control oral mucosa samples (CTR).

Sample		Alcian Blue	PAS	Picrosirius Red	Type-I Collagen
Control oral mucosa	CTR	100 ± 26.01	100 ± 27.38	100 ± 42.28	100 ± 39.4
Global groups	AFAS	23 ± 1.35	16.34 ± 1.94	12.11 ± 6.04	22.05 ± 1.45
	n-FAOM	21.22 ± 2.72	19.84 ± 10.42	19.66 ± 11.06	30.93 ± 5.85
	F-FAOM	41.79 ± 13.44	54.51 ± 23.97	55.79 ± 31.03	36.16 ± 17.33
Specific samples	AFAS-1W	22.87 ± 1.1	15.61 ± 0.79	6.04 ± 0.71	22.03 ± 0.48
	AFAS-2W	22.7 ± 1.14	16.71 ± 2	14.83 ± 2.76	22.11 ± 1.71
	AFAS-3W	23.4 ± 1.81	16.71 ± 2.63	15.47 ± 3.82	22.03 ± 1.89
	n-FAOM-1W	17.91 ± 1.2	21.1 ± 10.71	11.68 ± 8.1	28.64 ± 4.18
	n-FAOM-2W	18.97 ± 1.7	22.32 ± 5.21	20.95 ± 10.48	30.99 ± 4.19
	n-FAOM-3W	26.77 ± 1.73	16.1 ± 13.85	26.35 ± 5.54	33.17 ± 7.69
	F-FAOM-1W	17.55 ± 0.74	21.1 ± 10.04	29.81 ± 6.41	21.86 ± 4.23
	F-FAOM-2W	39.72 ± 4.4	55.61 ± 10.63	51.57 ± 9.26	34.17 ± 12.36
	F-FAOM-3W	68.09 ± 10.42	86.83 ± 4.64	85.98 ± 15.2	52.43 ± 6.9

## Supplementary Materials

The following are available online at https://www.mdpi.com/1996-1944/13/7/1692/s1, Table S1: Statistical comparison of staining intensity for histochemical and immunohistochemical analyses in the specific samples considered in the present study.

## References

[1] Witten C.M., McFarland R.D., Simek S.L. (2015). Concise Review: The US Food and Drug Administration and Regenerative Medicine. Stem Cells Transl. Med..

[2] Fagogeni I., Metlerska J., Lipski M., Falgowski T., Maciej G., Nowicka A. (2019). Materials used in regenerative endodontic procedures and their impact on tooth discoloration. J. Oral Sci..

[3] Larsson L., Decker A.M., Nibali L., Pilipchuk S.P., Berglundh T., Giannobile W.V. (2016). Regenerative Medicine for Periodontal and Peri-implant Diseases. J. Dent. Res..

[4] Langer R., Vacanti J.P. (1993). Tissue engineering. Science.

[5] Garzon I., Sanchez-Quevedo M.C., Moreu G., Gonzalez-Jaranay M., Gonzalez-Andrades M., Montalvo A., Campos A., Alaminos M. (2009). In vitro and in vivo cytokeratin patterns of expression in bioengineered human periodontal mucosa. J. Periodontal Res..

[6] Rana D., Ramasamy K., Leena M., Pasricha R., Manivasagam G., Ramalingam M., Vishwakarma A., Karp J.M. (2017). Surface functionalization of biomaterials. Biology and Engineering of Stem Cell Niches.

[7] Taraballi F., Zanini S., Lupo C., Panseri S., Cunha C., Riccardi C., Marcacci M., Campione M., Cipolla L. (2013). Amino and carboxyl plasma functionalization of collagen films for tissue engineering applications. J. Colloid Interface Sci..

[8] Stadlinger B., Hintze V., Bierbaum S., Moller S., Schulz M.C., Mai R., Kuhlisch E., Heinemann S., Scharnweber D., Schnabelrauch M. (2012). Biological functionalization of dental implants with collagen and glycosaminoglycans-A comparative study. J. Biomed. Mater. Res. B Appl. Biomater..

[9] Sapudom J., Rubner S., Martin S., Thoenes S., Anderegg U., Pompe T. (2015). The interplay of fibronectin functionalization and TGF-beta1 presence on fibroblast proliferation, differentiation and migration in 3D matrices. Biomater. Sci..

[10] Koh H.S., Yong T., Chan C.K., Ramakrishna S. (2008). Enhancement of neurite outgrowth using nano-structured scaffolds coupled with laminin. Biomaterials.

[11] Rosellini E., Cristallini C., Guerra G.D., Barbani N. (2015). Surface chemical immobilization of bioactive peptides on synthetic polymers for cardiac tissue engineering. J. Biomater. Sci. Polym. Ed..

[12] Rana D., Ramasamy K., Leena M., Jimenez C., Campos J., Ibarra P., Haidar Z.S., Ramalingam M. (2016). Surface functionalization of nanobiomaterials for application in stem cell culture, tissue engineering, and regenerative medicine. Biotechnol Prog.

[13] Martin-Piedra M.A., Alaminos M., Fernandez-Valades-Gamez R., Espana-Lopez A., Liceras-Liceras E., Sanchez-Montesinos I., Martinez-Plaza A., Sanchez-Quevedo M.C., Fernandez-Valades R., Garzon I. (2017). Development of a multilayered palate substitute in rabbits: A histochemical ex vivo and in vivo analysis. Histochem. Cell Biol..

[14] Garzon I., Miyake J., Gonzalez-Andrades M., Carmona R., Carda C., Sanchez-Quevedo Mdel C., Campos A., Alaminos M. (2013). Wharton’s jelly stem cells: A novel cell source for oral mucosa and skin epithelia regeneration. Stem Cells Transl Med.

[15] Garzon I., Martin-Piedra M.A., Alfonso-Rodriguez C., Gonzalez-Andrades M., Carriel V., Martinez-Gomez C., Campos A., Alaminos M. (2014). Generation of a biomimetic human artificial cornea model using Wharton’s jelly mesenchymal stem cells. Investig. Ophthalmol. Vis. Sci..

[16] Scionti G., Moral M., Toledano M., Osorio R., Duran J.D., Alaminos M., Campos A., Lopez-Lopez M.T. (2014). Effect of the hydration on the biomechanical properties in a fibrin-agarose tissue-like model. J. Biomed. Mater. Res. A.

[17] Alfonso-Rodriguez C.A., Gonzalez-Andrades E., Jaimes-Parra B.D., Fernandez-Valades R., Campos A., Sanchez-Quevedo M.C., Alaminos M., Garzon I. (2015). Ex vivo and in vivo modulatory effects of umbilical cord Wharton’s jelly stem cells on human oral mucosa stroma substitutes. Histol. Histopathol..

[18] Vinuela-Prieto J.M., Sanchez-Quevedo M.C., Alfonso-Rodriguez C.A., Oliveira A.C., Scionti G., Martin-Piedra M.A., Moreu G., Campos A., Alaminos M., Garzon I. (2015). Sequential keratinocytic differentiation and maturation in a three-dimensional model of human artificial oral mucosa. J. Periodontal Res..

[19] Oliveira A.C., Garzon I., Ionescu A.M., Carriel V., Cardona Jde L., Gonzalez-Andrades M., Perez Mdel M., Alaminos M., Campos A. (2013). Evaluation of small intestine grafts decellularization methods for corneal tissue engineering. PLoS ONE.

[20] Fernandez-Valades-Gamez R., Garzon I., Liceras-Liceras E., Espana-Lopez A., Carriel V., Martin-Piedra M.A., Munoz-Miguelsanz M.A., Sanchez-Quevedo M.C., Alaminos M., Fernandez-Valades R. (2016). Usefulness of a bioengineered oral mucosa model for preventing palate bone alterations in rabbits with a mucoperiostial defect. Biomed. Mater..

[21] Martin-Piedra M.A., Garzon I., Gomez-Sotelo A., Garcia-Abril E., Jaimes-Parra B.D., Lopez-Cantarero M., Alaminos M., Campos A. (2017). Generation and Evaluation of Novel Stromal Cell-Containing Tissue Engineered Artificial Stromas for the Surgical Repair of Abdominal Defects. Biotechnol. J..

[22] Egea-Guerrero J.J., Carmona G., Correa E., Mata R., Arias-Santiago S., Alaminos M., Gacto P., Cuende N. (2019). Transplant of Tissue-Engineered Artificial Autologous Human Skin in Andalusia: An Example of Coordination and Institutional Collaboration. Transpl. Proc..

[23] Gonzalez-Andrades M., Mata R., Gonzalez-Gallardo M.D.C., Medialdea S., Arias-Santiago S., Martinez-Atienza J., Ruiz-Garcia A., Perez-Fajardo L., Lizana-Moreno A., Garzon I. (2017). A study protocol for a multicentre randomised clinical trial evaluating the safety and feasibility of a bioengineered human allogeneic nanostructured anterior cornea in patients with advanced corneal trophic ulcers refractory to conventional treatment. BMJ Open.

[24] Moussa D.G., Aparicio C. (2019). Present and future of tissue engineering scaffolds for dentin-pulp complex regeneration. J. Tissue Eng. Regen. Med..

[25] Kasai Y., Takagi R., Kobayashi S., Owaki T., Yamaguchi N., Fukuda H., Sakai Y., Sumita Y., Kanai N., Isomoto H. (2020). A stable protocol for the fabrication of transplantable human oral mucosal epithelial cell sheets for clinical application. Regen. Ther..

[26] Heathman T.R., Glyn V.A., Picken A., Rafiq Q.A., Coopman K., Nienow A.W., Kara B., Hewitt C.J. (2015). Expansion, harvest and cryopreservation of human mesenchymal stem cells in a serum-free microcarrier process. Biotechnol. Bioeng..

[27] Costa A., Naranjo J.D., Londono R., Badylak S.F. (2017). Biologic Scaffolds. Cold Spring Harb. Perspect. Med..

[28] Halper J., Kjaer M. (2014). Basic components of connective tissues and extracellular matrix: Elastin, fibrillin, fibulins, fibrinogen, fibronectin, laminin, tenascins and thrombospondins. Adv. Exp. Med. Biol..

[29] Aviezer D., Levy E., Safran M., Svahn C., Buddecke E., Schmidt A., David G., Vlodavsky I., Yayon A. (1994). Differential structural requirements of heparin and heparan sulfate proteoglycans that promote binding of basic fibroblast growth factor to its receptor. J. Biol. Chem..

[30] Browning K.N. (2018). Extracellular matrix proteins in the gastrointestinal tract: More than a supporting role. J. Physiol..

[31] Gandolfi M.G., Gardin C., Zamparini F., Ferroni L., Esposti M.D., Parchi G., Ercan B., Manzoli L., Fava F., Fabbri P. (2020). Mineral-Doped Poly(L-lactide) Acid Scaffolds Enriched with Exosomes Improve Osteogenic Commitment of Human Adipose-Derived Mesenchymal Stem Cells. Nanomaterials.

[32] Kubo K. (2018). Effects of static stretching on mechanical properties and collagen fiber orientation of the Achilles tendon in vivo. Clin. Biomech..

[33] Osorio R., Alfonso-Rodriguez C.A., Osorio E., Medina-Castillo A.L., Alaminos M., Toledano-Osorio M., Toledano M. (2017). Novel potential scaffold for periodontal tissue engineering. Clin. Oral Investig..

