# Erratum: Blanco-Elices, C. et al. In Vitro Generation of Novel Functionalized Biomaterials for Use in Oral and Dental Regenerative Medicine Applications. Running Title: Fibrin–Agarose Functionalized Scaffolds. *Materials* 2020, *13*, 1692

**DOI:** 10.3390/ma13225205

**Published:** 2020-11-18

**Authors:** Cristina Blanco-Elices, Enrique España-Guerrero, Miguel Mateu-Sanz, David Sánchez-Porras, Óscar Darío García-García, María del Carmen Sánchez-Quevedo, Ricardo Fernández-Valadés, Miguel Alaminos, Miguel Ángel Martín-Piedra, Ingrid Garzón

**Affiliations:** 1 Department of Histology (Tissue Engineering Group), University of Granada, 18071 Granada, Spain; acblanco96@gmail.com (C.B.-E.); miguel.mateu@upc.edu (M.M.-S.); davidsp94@correo.ugr.es (D.S.-P.); oscargg@correo.ugr.es (Ó.D.G.-G.); mcsanchez@ugr.es (M.d.C.S.-Q.); rfdezvalades@me.com (R.F.-V.); malaminos@ugr.es (M.A.); 2 Programa de Doctorado Medicina Clínica y Salud Pública, University of Granada, 18071 Granada, Spain; enrique@dentalos.es; 3 Department Materials Science and Metallurgy (Biomaterials, Biomechanics and Tissue Engineering Group), Technical University of Catalonia, 08019 Barcelona, Spain; 4 Department of Histology, Faculty of Medicine, University of Granada, Avenida de la Investigación 11, E18016, 18071 Granada, Spain; igarzon@ugr.es

## Incorrect Title

There is an error in the title. The correct title of the paper [1] is ‘Blanco-Elices, C. In Vitro Generation of Novel Functionalized Biomaterials for Use in Oral and Dental Regenerative Medicine Applications.’ We apologize for this error and state that the scientific conclusions are unaffected. The original article has been updated. 
