# Supplementary material for: In Vitro Generation of Novel Functionalized Biomaterials for Use in Oral and Dental Regenerative Medicine Applications. Running Title: Fibrin–Agarose Functionalized Scaffolds
Source: Materials (Basel). 2020 Apr 4;13(7):1692. doi: 10.3390/ma13071692 (PMC7178710; doi:10.3390/ma13071692)
Supplement: Supplementary file 1 [file materials-13-01692-s001.pdf]

materials

MDPI

Supplementary Materials

In Vitro Generation of Novel Functionalized Biomaterials for Use in Oral and Dental Regenerative Medicine Applications. Running Title: Fibrin–Agarose Functionalized Scaffolds

Table S1. Statistical comparison of staining intensity for histochemical and immunohistochemical analyses in the specific samples considered in the present study.

		AFAS-1W	AFAS-2W	AFAS-3W	n-FAOM-1W	n-FAOM-2W	n-FAOM-3W	F-FAOM-1W	F-FAOM-2W	F-FAOM-3W	CTR
AB	AFAS-1W	-	1.0000	1.0000	0.9995	0.9999	0.9999	0.9991	0.3690	**<0.0001***	**<0.0001***
	AFAS-2W	1.0000	-	1.0000	0.9996	1.0000	0.9999	0.9993	0.3541	**<0.0001***	**<0.0001***
	AFAS-3W	1.0000	1.0000	-	0.9989	0.9998	1.0000	0.9982	0.4156	**<0.0001***	**<0.0001***
	n-FAOM-1W	0.9995	0.9996	0.9989	-	1.0000	0.9644	1.0000	0.0856	**<0.0001***	**<0.0001***
	n-FAOM-2W	0.9999	1.0000	0.9998	1.0000	-	0.9847	1.0000	0.1230	**<0.0001***	**<0.0001***
	n-FAOM-3W	0.9999	0.9999	1.0000	0.9644	0.9847	-	0.9544	0.7305	**<0.0001***	**<0.0001***
	F-FAOM-1W	0.9991	0.9993	0.9982	1.0000	1.0000	0.9544	-	0.0754	**<0.0001***	**<0.0001***
	F-FAOM-2W	0.3690	0.3541	0.4156	0.0856	0.1230	0.7305	0.0754	-	**0.0056****	**<0.0001***
	F-FAOM-3W	**<0.0001***	**<0.0001***	**<0.000***	**<0.0001***	**<0.0001***	**<0.0001***	**<0.0001***	**0.0056****	-	**0.0010****
	CTR	**<0.0001***	**<0.0001***	**<0.0001***	**<0.0001***	**<0.0001***	**<0.0001***	**<0.0001***	**<0.0001***	**0.0010****	-
PAS	AFAS-1W	-	1.0000	1.0000	0.9969	0.9867	1.0000	0.9969	**<0.0001***	**<0.0001***	**<0.0001***
	AFAS-2W	1.0000	-	1.0000	0.9995	0.9964	1.0000	0.9995	**<0.0001***	**<0.0001***	**<0.0001***
	AFAS-3W	1.0000	1.0000	-	0.9995	0.9964	1.0000	0.9995	**<0.0001***	**<0.0001***	**<0.0001***
	n-FAOM-1W	0.9969	0.9995	0.9995	-	1.0000	0.9985	1.0000	**<0.0001***	**<0.0001***	**<0.0001***
	n-FAOM-2W	0.9867	0.9964	0.9964	1.0000	-	0.9922	1.0000	**<0.0001***	**<0.0001***	**<0.0001***
	n-FAOM-3W	1.0000	1.0000	1.0000	0.9985	0.9922	-	0.9985	**<0.0001***	**<0.0001***	**<0.0001***
	F-FAOM-1W	0.9969	0.9995	0.9995	1.0000	1.0000	0.9985	-	**<0.0001***	**<0.0001***	**<0.0001***
	F-FAOM-2W	**<0.0001***	**<0.0001***	**<0.0001***	**<0.0001***	**<0.0001***	**<0.0001***	**<0.0001***	-	**0.0001**	**<0.0001***
	F-FAOM-3W	**<0.0001***	**<0.0001***	**<0.0001***	**<0.0001***	**<0.0001***	**<0.0001***	**<0.0001***	**0.0001***	-	0.5366
	CTR	**<0.0001***	**<0.0001***	**<0.0001***	**<0.0001***	**<0.0001***	**<0.0001***	**<0.0001***	**<0.0001***	0.5366	-
PICROS	AFAS-1W	-	0.8534	0.7938	0.9906	0.1960	**0.0149**	**0.0018****	**<0.0001***	**<0.0001***	**<0.0001***
	AFAS-2W	0.8534	-	1.0000	0.9999	0.9832	0.5496	0.1900	**<0.0001***	**<0.0001***	**<0.0001***
	AFAS-3W	0.7938	1.0000	-	0.9996	0.9923	0.6294	0.2411	**<0.0001***	**<0.0001***	**<0.0001***

	**n-FAOM-1W**	0.9906	0.9999	0.9996	-	0.8097	0.2145	0.0473**	<0.0001*	<0.0001*	<0.0001*
	**n-FAOM-2W**	0.1960	0.9832	0.9923	0.8097	-	0.9931	0.8466	<0.0001*	<0.0001*	<0.0001*
	**n-FAOM-3W**	0.0149**	0.5496	0.6294	0.2145	0.9931	-	0.9998	0.0007**	<0.0001*	<0.0001*
	**F-FAOM-1W**	0.0018**	0.1900	0.2411	0.0473**	0.8466	0.9998	-	0.0064**	<0.0001*	<0.0001*
	**F-FAOM-2W**	<0.0001*	<0.0001*	<0.0001*	<0.0001*	<0.0001*	0.0007**	0.0064**	-	<0.0001*	<0.0001*
	**F-FAOM-3W**	<0.0001*	<0.0001*	<0.0001*	<0.0001*	<0.0001*	<0.0001*	<0.0001*	<0.0001*	-	0.2698
	**CTR**	<0.0001*	<0.0001*	<0.0001*	<0.0001*	<0.0001*	<0.0001*	<0.0001*	<0.0001*	0.2698	-
**COLI-IHC**	**AFAS-1W**	-	1.0000	1.0000	0.9532	0.7644	0.4820	1.0000	0.3561	<0.0001*	<0.0001*
	**AFAS-2W**	1.0000	-	1.0000	0.9567	0.7740	0.4931	1.0000	0.3660	<0.0001*	<0.0001*
	**AFAS-3W**	1.0000	1.0000	-	0.9532	0.7644	0.4820	1.0000	0.3561	<0.0001*	<0.0001*
	**n-FAOM-1W**	0.9532	0.9567	0.9532	-	1.0000	0.9966	0.9454	0.9856	0.0005**	<0.0001*
	**n-FAOM-2W**	0.7644	0.7740	0.7644	1.0000	-	1.0000	0.7448	0.9998	0.0026**	<0.0001*
	**n-FAOM-3W**	0.4820	0.4931	0.4820	0.9966	1.0000	-	0.4601	1.0000	0.0107**	<0.0001*
	**F-FAOM-1W**	1.0000	1.0000	1.0000	0.9454	0.7448	0.4601	-	0.3368	<0.0001*	<0.0001*
	**F-FAOM-2W**	0.3561	0.3660	0.3561	0.9856	0.9998	1.0000	0.3368	-	0.0198**	<0.0001*
	**F-FAOM-3W**	<0.0001*	<0.0001*	<0.0001*	0.0005**	0.0026**	0.0107**	<0.0001*	0.0198**	-	<0.0001*
	**CTR**	<0.0001*	<0.0001*	<0.0001*	<0.0001*	<0.0001*	<0.0001*	<0.0001*	<0.0001*	<0.0001*	-
		* p < 0.0001									
		** p < 0.05									

Values correspond to P-values, and statistically significant values are labeled with one asterisk (*), whereas marginally-significant values are labeled with two asterisks (**). Alcian Blue (AB), Periodic acid- Schiff (PAS), Picrosirius (PICROS) staining and Collagen type I immunostaining (COLI-IHC).
